# Development of colorectal cancer predicts increased risk of subsequent hepatocellular carcinoma in patients with alcoholic liver disease: case-control and cohort study

**DOI:** 10.1038/s41598-019-39573-9

**Published:** 2019-03-01

**Authors:** Won Kim, Dongjae Jeong, Jungwha Chung, Donghyeon Lee, Saekyoung Joo, Eun Sun Jang, Yoon Jin Choi, Hyuk Yoon, Cheol Min Shin, Young Soo Park, Sook-Hyang Jeong, Nayoung Kim, Dong Ho Lee, Jin-Wook Kim

**Affiliations:** 1 Department of Medicine, Seoul National University College of Medicine, Seoul, Republic of Korea; 2 Department of Medicine, Seoul National University Bundang Hospital, Seongnam, Republic of Korea; 3 Department of Internal Medicine, Seoul Metropolitan Government Seoul National University Boramae Medical Center, Seoul, Republic of Korea

## Abstract

Alcohol increases the risk of both hepatocellular carcinoma (HCC) and colorectal neoplasia. In this hospital-based case-control and retrospective cohort study, we sought to determine whether development of colorectal neoplasia increases the risk of HCC in patients with alcoholic liver disease (ALD). In the phase I case-control analysis, the association between history of colorectal cancer (CRC) and HCC development was assessed in patients with ALD by logistic regression modeling (n = 1,659). In the phase II retrospective cohort analysis, the relative risk of HCC development was compared in ALD patients with respect to the history of CRC by a Cox model (n = 1,184). The history of CRC was significantly associated with HCC in the case-control analysis (adjusted odds ratio, 1.82; 95% CI, 1.06–3.15; *P* < 0.05). ALD patients with CRC had higher risk of developing HCC compared to those without CRC (adjusted hazards ratio [HR], 5.48; 95% CI, 1.63–18.36; *P* = 0.006) in the cohort analysis. Presence of CRC, liver cirrhosis, elevated baseline alpha-fetoprotein level, and low platelet counts were independent predictors of HCC development in ALD patients. Patients with history of CRC had an increased risk of HCC in both cirrhotic (HR, 3.76; 95% CI, 1.05–13.34, *P* = 0.041) and non-cirrhotic (HR, 23.46; 95% CI, 2.81–195.83, *P* = 0.004) ALD patients. In conclusion, ALD patients with CRC are at increased risk of developing HCC.

## Introduction

Alcohol-related liver disease is a global health burden that causes 348,000 deaths and 10,997,000 disability-adjusted life years annually^1^. Alcohol causes a spectrum of liver diseases comprising simple steatosis, alcoholic hepatitis, cirrhosis, and hepatocellular carcinoma (HCC)^2,3^. Alcohol is the second most important risk factor for HCC, following viral hepatitis^4^, and alcohol-related HCC deaths have not significantly decreased over the last decade^5^. Early detection is critical to improve the prognosis of HCC, but no effective strategies have been established for the early diagnosis of HCC in patients with alcoholic liver disease (ALD)^4,6,7^. Cirrhosis is one of the most important predictors for alcohol-related HCC, along with old age, comorbid chronic viral hepatitis, and the amount of alcohol consumption^8–10^. However, even patients with alcoholic cirrhosis have relatively lower risk of developing HCC compared to those with viral hepatitis-associated cirrhosis^11–13^. Therefore, further refined risk stratification is prerequisite for the development of a cost-effective HCC surveillance strategy in patients with ALD.

Alcohol is a risk factor not only for HCC but also for colorectal neoplasm: excessive alcohol consumption increases the likelihood of developing adenomas and colorectal cancer (CRC)^14–17^. Chronic ethanol consumption usually promotes carcinogenesis by production of toxic acetaldehyde, induction of cytochrome P450 2E1 that causes oxidative stress and procarcinogen activation, and provocation of global DNA hypomethylation^18^. Since these carcinogenic pathways are known to contribute to the development of both colorectal neoplasm and HCC^10,19,20^, we hypothesized that ALD patients who develop colorectal neoplasm are at risk of further developing HCC. The aim of this study was to elucidate the relationship between the presence of colorectal neoplasm and the subsequent risk of HCC in patients with ALD.

## Methods

### Data sources

We recruited ALD patients from two university-affiliated, tertiary referral centers in South Korea. Patients with ALD were identified from the electronic health record of the centers^21,22^ by the World Health Organization International Classification of Diseases, tenth revision (ICD-10) code of K70. The diagnosis of alcoholic liver disease was made by four hepatologists (WK, ESJ, SHJ and JWK) by documentation of alcoholic excess and evidence of liver disease^23^. Alcohol consumption amount was assessed via the hepatologists’ interview with patients and their family members. Diagnosis of fatty liver was made radiologically^24^, and liver cirrhosis was diagnosed by liver biopsy, radiological findings or presence of esophageal varix^25^. Clinical investigations were conducted according to the principles expressed in the Declaration of Helsinki^26^, and the requirement for informed consent was waived due to the retrospective nature of the current study and anonymous analysis of the data. The institutional review board approved this study (Seoul National University Bundang Hospital IRB; IRB no. B-1703/388-108).

### Study design

**Figure 1:**
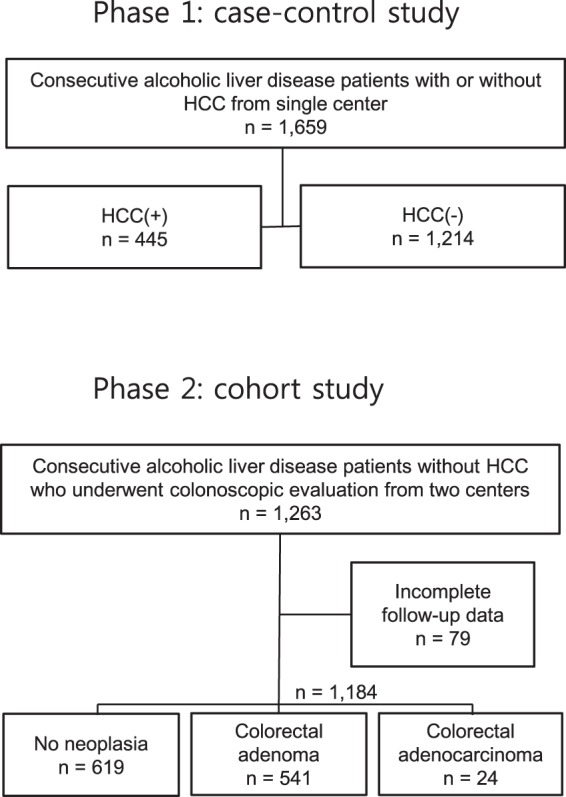
Study flow and attrition diagram of study participants.

This study consists of two-phase investigations: phase I case-control analysis and phase II retrospective cohort analysis (Fig. 1). The case-control analysis aimed to investigate the possible association between history of CRC and HCC risk in ALD patients, whereas the cohort analysis sought to determine the relative risk of HCC in ALD patients with the history of colorectal adenoma (CRA) or CRC.

### Phase I case-control analysis

In the phase I single-center case-control analysis, we selected 1,659 ALD patients who received imaging work-up for HCC, i.e., liver ultrasound (US), computed tomography (CT) or magnetic resonance imaging (MRI) at Seoul National University Bundang Hospital between April, 2003 and April, 2018. Patients with evidence of other chronic liver disease such as hepatitis B or C virus infection, hemochromatosis, Wilson disease, primary biliary cholangitis, autoimmune hepatitis, and primary sclerosing cholangitis were excluded. Decompensated patients with Child-Pugh class C were also excluded from analysis.

ALD patients diagnosed with HCC were selected as cases, and those without any evidence of HCC were selected as controls. The index date was defined as the date of HCC diagnosis for cases, and the date of initial imaging work-up for controls. The history of CRC was coded positive if a patient had received surgical treatment for CRC or if colonoscopic examination(s), performed as described below, revealed colorectal adenocarcinoma before the index date.

### Phase II cohort analysis

In the phase II retrospective cohort analysis, an ALD cohort was electronically formed by assembling ALD patients who received imaging work-up for HCC *and* colonoscopic examination(s) from the two centers. The cohort was composed of a subgroup of the phase I controls and a separate cohort formed at Boramae Medical Center. Patients with evidence of other chronic liver disease were excluded in the same way as the phase I cohort, and the following additional exclusion criteria were applied: patients were excluded if HCC was detected at initial screening or within 6 months after initial screening or if follow-up data was incomplete. The observation starting point for the longitudinal cohort analysis was the time of colonoscopic examination(s). During follow-up, patients underwent additional liver US with or without alpha-fetoprotein (AFP) levels. Follow-up US at 6 months of interval was recommended for patients with alcoholic liver cirrhosis, and follow-up US was performed at the discretion of attending hepatologists for patients without evidence of liver cirrhosis. Dynamic CT or MRI was performed if serum AFP levels or surveillance US indicated a need for further evaluation according to the recall policies^27,28^. Liver biopsy was performed if CT or MRI showed new hepatic nodule(s) which did not show typical enhancement pattern diagnostic of HCC. The primary outcome of this study was the incidence of HCC, which was diagnosed based on histological or radiological findings^29^.

### CRC screening in the study population

CRC is the third most common cancer in Korea^30^, and Korean National Cancer Screening Programme has implemented programmatic CRC screening by means of fecal occult blood tests for all Koreans aged ≥ 50 years since 2004^31^. If the fecal occult blood test is positive, colonoscopy or double-contrast barium enema has been provided. Colonoscopy has also been recommended for CRC screening regardless of the occult blood test result in average-risk asymptomatic adults according to clinicians’ discretion and recipients’ preference^32^. In our cohorts, colonoscopic CRC screening was performed either on the programmatic basis following the Korean guideline or on the opportunistic basis. If colonoscopic examinations were performed more than once, the highest grade of colonic pathology was determined and the earliest date of examination was chosen as the ultimate grade of pathology.

### Statistical analysis

Statistical analysis was performed using STATA version 14 (Stata Corp, College Station, TX) and R version 3.4.2 (http://www.cran.r-project.org/). Wilcoxon rank sum test or one-way ANOVA test were used for continuous variables, and chi-square test was used to analyze categorical variables. In phase I case-control analysis, the odds ratios (ORs) for HCC were calculated by logistic regression analysis. In phase II cohort analysis, the cumulative incidence of HCC was calculated using the Kaplan-Meier method and the hazard ratios (HRs) for HCC were derived by a Cox proportional hazards model.

## Results

### Phase I case-control analysis: association between the history of CRC and the risk of HCC in patients with ALD

The case-control analysis compared 445 ALD-related HCC cases and 1,214 ALD controls without HCC with respect to the history of CRC. Of the 445 HCC patients, 24 had history of CRC, among whom 7 HCC was confirmed pathologically. HCC patients showed older age, higher proportion of female sex, higher smoking rate and drinking amount, higher probability of the history of CRC (5.4% vs. 2.7%; p = 0.008) (Table 1). Logistic regression analysis showed that past history of CRC was significantly associated with future development of HCC (unadjusted OR, 2.04; 95% CI, 1.19–3.49; *P* < 0.001), along with sex, drinking amount and smoking as additional significant variables associated with HCC development (Table 2). The association of CRC history with HCC remained significant after adjustment for confounders (adjusted OR, 1.82; 95% CI, 1.06–3.15; *P* < 0.05), indicating possible association between development of CRC and HCC in ALD.

**Table 1:** Case-control analysis: characteristics of alcoholic liver disease patients with and without hepatocellular carcinoma.

	ALD patients with HCC cases (n = 445)	Control ALD patients without HCC (n = 1,214)	*P* value
Age, years	64 (15)	49 (14)	**<0**.**001**
Male	410 (92)	1,158 (95)	**0**.**01**
Drinking amount (>80 g/day of ethanol)	315 (71)	710 (58)	<0.001
Current or ex-smoker	444 (99)	1,166 (96)	**<0**.**001**
Prior history of CRC	24 (5.4)	33 (2.7)	**0**.**008**
AFP (ng/mL)	5.4 (23.3)	3.0 (1.9)	**<0**.**001**
Albumin (g/dL)	3.8 (1.0)	4.5 (0.5)	**<0**.**001**
Bilirubin (mg/dL)	1.1 (1.0)	1.1 (0.6)	0.481
AST (U/L)	48 (73)	35 (27)	**<0**.**001**
ALT (U/L)	32 (31)	41 (39)	**<0**.**001**
GGT (U/L)	121 (205)	77 (107)	**<0**.**001**
Platelet count, x10^9^/L	162 (121)	224 (76)	**<0**.**001**
Prothrombin time (INR)	1.10 (0.25)	1.01 (0.09)	**<0**.**001**

Continuous variables are expressed as the median (interquartile range) and categorical variables are presented as numbers (%).

Laboratory data were values at the index date (confirmation of presence/absence of HCC).

*P* values were calculated by Wilcoxon rank sum test or χ^2^ test for continuous and categorical variables, respectively.

CRC, colorectal cancer.

**Table 2:** Odds ratio for HCC in the phase I case-control analysis (n = 1,659).

	Unadjusted OR (95% CI)	Adjusted OR (95% CI)
Age (>35 years)	3.44 (1.65–7.21)**	3.46 (1.64–7.30)**
Male	**0**.**57** (**0**.**37–0**.**88**)*	0.54 (0.35–0.85)**
Drinking amount (>80 g ethanol/day)	**1**.**72** (**1**.**36–2**.**17**)*	1.73 (1.36–2.19)*
Current or ex-smoker	**18**.**3** (**2**.**52–132**.**8**)*	18.5 (2.53–135.1)**
Prior history of CRC	**2**.**04** (**1**.**19–3**.**49**)*	1.82 (1.06–3.15)**

*P < 0.001, **P < 0.05

CRC, colorectal cancer; OR, odds ratio.

### Phase II cohort analysis: presence of CRC as an independent predictor of future HCC development in ALD

A total of 1,263 patients were assembled for the longitudinal cohort. After excluding 79 patients with incomplete follow-up information, 1,184 patients were finally selected for the cohort analysis (Fig. 1). CRA and CRC were present in 46% and 2% of ALD cohort, respectively, at the baseline screening colonoscopic examination (Table 3). One-third of CRC patients were diagnosed at the stage of carcinoma *in situ* (T*is*, n = 8/24). Patients with CRC showed higher age, higher proportion of cirrhosis with worse hepatic reserve, higher frequency of smoking and greater amount of alcohol consumption.

**Table 3:** Baseline characteristics of ALD patients in the phase II cohort study (n = 1,184).

	Without colonic neoplasia (n = 619)	With CRA (n = 541)	With CRC (n = 24)	*P* value
Age, years	53 (15)	54 (14)	64 (13)	<0.001
Male, n (%)	578 (93)	526 (97)	15 (92)	0.007
Liver cirrhosis, n (%)	98 (16)	113 (21)	16 (67)	<0.001
alcohol intake amount (g/day)	57 (70)	69 (69)	108 (81)	0.007
Current or ex-smoker, n (%)	431 (74)	427 (81)	23 (96)	0.002
AFP (ng/mL)	3.0 (1.2)	3.0 (1.9)	3.9 (1.8)	0.647
Albumin (g/dL)	4.4 (0.5)	4.4 (0.6)	3.6 (1.0)	<0.001
Bilirubin (mg/dL)	1.1 (0.6)	1.1 (0.6)C	1.0 (0.6)	0.777
AST (U/L)	33 (26)	34 (25)	30 (17)	0.372
ALT (U/L)	35 (34)	36 (31)	22 (16)	0.023
GGT (U/L)	100 (92)	103 (98)	104 (74)	0.587
Platelet count, x10^9^/L	219 (75)	222 (83)	150 (79)	0.006
Prothrombin time (INR)	1.02 (0.10)	1.01 (0.09)	1.07 (0.23)	0.005
CRC T stage (Tis/1/2/3/4/x)			8/3/2/9/1/1	

Continuous variables are expressed as the median (interquartile range) and categorical variables are presented as numbers (%).

*P* values were calculated by one-way ANOVA test and χ^2^ test for continuous and categorical variables, respectively.

AFP, Alpha-fetoprotein; AST, aspartate aminotransferase; ALT, alanine aminotransferase; CRA, colorectal adenoma; CRC, colorectal cancer; GGT, gamma-glutamyl transferase; INR, international normalized ratio.

**Figure 2:**
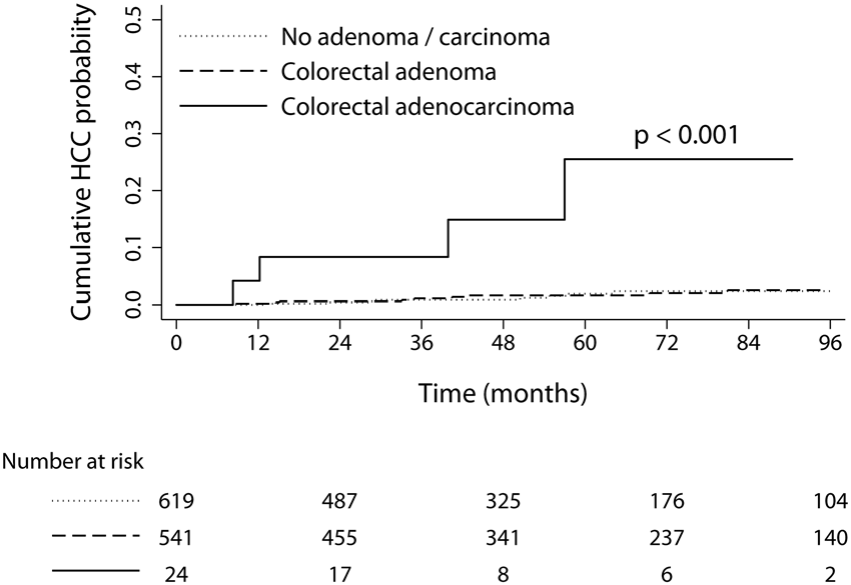
Incidence of HCC according to the colonoscopic histopathology in the phase II cohort study. Colorectal adenocarcinoma includes carcinoma *in situ*.

During the median follow-up of 55 months (interquartile range [IQR] = 60, 72,117 person-years), 24 patients developed HCC with the incidence of 33.3/100,000 person-years (95% CI, 22.3–49.7). Among the 24 patients, 4 had history of CRC and HCC was pathologically confirmed in all 4 patients. When the cumulative HCC incidences were compared according to colonic pathologic findings, patients with CRC showed significantly higher probability of further developing HCC compared to patients without colonic neoplasia or patients with CRA (Fig. 2). The median (IQR) size of HCC was not significantly different between patients with and without preceding CRC: 2.7 (5.6) cm vs. 1.9 (3.8) cm for patients with vs. without preceding CRC, respectively (*P* = 0.312 by Wilcox rank sum test).

**Figure 3:**
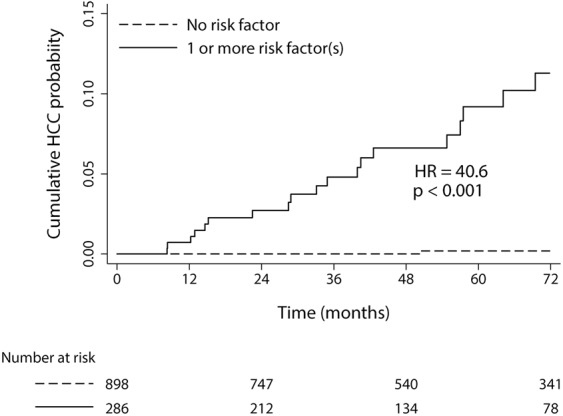
Incidence of HCC according to the presence or absence of risk factors in the phase II cohort analysis.

Univariate Cox regression analysis showed that patients with CRC had higher risk of developing HCC compared to those without CRC (HR, 12.64; 95% CI, 4. 28–37.15; *P* < 0.001). CRA was not significantly associated with the risk of HCC development (HR, 0.92; 95% CI, 0.38–2.21; *P* = 0.851) (Table 4). Other significant baseline risk factors for HCC development included old age, presence of liver cirrhosis, high in alcohol consumption amount, high AFP level, low albumin level, prolonged prothrombin time, and low platelet counts. CRC remained as an independent predictor of HCC development in multivariate analysis (HR, 5.48; 95% CI, 1.63–18.36; *P* = 0.006), along with liver cirrhosis (HR, 3.23; 95% CI, 1.22–8.63; *P* = 0.018), AFP level > 10 ng/mL (HR, 4.26; 95% CI, 1.41–12.85; *P* = 0.010), and platelet count < 125×10^9^/L (HR, 5.76; 95% CI, 2.19–15.11; *P* < 0.001). Patients with any of the risk factors had significantly higher incidence of HCC compared to patients without risk factor (HR, 40.60; 95% CI, 9.53–172.93; *P* < 0.001) (Fig. 3). History of CRC increased the HCC risk regardless of cirrhosis: the HR for HCC was 3.76 (95% CI, 1.05–13.34, *P* = 0.041) and 23.46 (95% CI, 2.81–195.83, *P* = 0.004) for ALD patients with or without liver cirrhosis, respectively.

**Table 4:** Predictors of incident HCC by the Cox proportional hazards model in the study cohort.

Parameters	Univariate			Multivariate		
	HR	95% CI	*P* value	HR	95% CI	*P* value
Age (>50 years)	4.64	1.34–25.56	**0**.**019**	2.24	0.64–7.89	0.209
Male sex	0.41	0.10–1.77	0.233			
Liver cirrhosis	11.36	4.71–27.44	**<0**.**001**	3.25	1.22–8.63	**0**.**018**
Alcohol intake (>80 g/day)	2.76	1.18–6.44	**0**.**019**	1.71	0.70–4.19	0.242
Current or ex-smoking	2.57	0.60–10.98	0.203			
AFP>10 ng/mL	8.74	2.90–26.31	**<0**.**001**	4.26	1.41 –12.85	**0**.**010**
Albumin <3.8 g/dL	6.96	3.02–16.05	**<0**.**001**	1.19	0.47–3.04	0.715
Bilirubin >1.5 mg/dL	1.00	0.29–3.48	0.997			
PT INR >1.2	8.83	3.73–20.90	**<0**.**001**	1.46	0.53–4.07	0.466
Platelet count < 125 × 10^9^/L	16.60	7.38–37.36	**<0**.**001**	5.76	2.19–15.11	**<0**.**001**
AST > 40 U/L	2.24	1.00–5.01	**0**.**049**	1.53	0.62–3.76	0.354
ALT > 40 U/L	0.94	0.42–2.13	0.885			
GGT > 60 U/L	3.42	0.98–11.90	0.053			
**Colorectal pathology**						
No neoplasia	1.00			1.00		
CRA	0.92	0.38–2.21	0.851			
CRC*	12.64	4.28–37.15	<**0**.**001**	5.48	1.63–18.36	**0**.**006**

*Adenocarcinoma vs. no neoplasia or adenoma. Colorectal adenocarcinoma includes carcinoma *in situ*.

AFP, Alpha-fetoprotein; AST, aspartate aminotransferase; ALT, alanine aminotransferase; CRA, colorectal adenoma; CRC, colorectal cancer; GGT, gamma-glutamyl transferase; INR, international normalized ratio.

## Discussion

Our study found that the risk of subsequent HCC development increased when CRCs were detected in patients with ALD. The initial case-control analysis revealed higher proportion of the prior history of CRC in HCC, suggesting association between the development of CRC and HCC in ALD. The Phase I case-control design cannot confirm the causal relationship, but Phase II longitudinal cohort analysis also demonstrated increased OR of HCC by the history of CRC. Taken together, we believe that the Phase I and II analyses indicate a significant association between the history of CRC and the risk of HCC. Several risk factors for HCC have been established in ALD^8,10^, but CRC has not been recognized as one yet. The association between CRC and subsequent HCC remained significant after adjusting for known covariates for HCC, indicating that presence of CRC has additional predictive power.

We did not include presence/absence of cirrhosis in Phase I logistic model because not all patients underwent histologic examinations or endoscopic varix study. Since thrombocytopenia is one of the clinical diagnostic criteria of cirrhosis, we believe that the significant difference in platelet counts in Phase I analysis indicate significant association of liver cirrhosis with development of HCC in ALD. Similarly, low albumin levels and platelet counts in the CRC group may be explained by the higher prevalence of liver cirrhosis in Phase II analysis (Table 3). However, detection of CRC remained significant in the multivariate Cox model (Table 4), indicating that CRC history may be an independent risk factor for further HCC irrespective of liver cirrhosis.

Our study did not provide mechanistic explanation of the association between HCC and preceding CRC. We speculate that presence of CRC may indicate either exposure to alcohol exceeding a certain threshold level, or increased host susceptibility to alcohol for malignancies for the following findings. First, the carcinogenic effect of alcohol and/or aldehyde metabolites on colonic mucosa might also contribute to the carcinogenesis of HCC^10,19,20^. Second, alcohol-induced changes in gut microbiota increase acetaldehyde production^33,34^, which is a common inducer of both cancers. Moreover, alcohol-induced dysbiosis increases the level of deoxycholic acid (DCA), a gut bacterial metabolite known as a hepatocarcinogen by inducing a senescence-associated secretory phenotype in hepatic stellate cells^35^. Colonic neoplasia as well as alcohol intake may enhance intestinal permeability and enterohepatic circulation of DCA, which in turn facilitate the development of HCC. Epigenetic^36^ or immunologic^37^ alterations by alcohol may also contribute to the pathogenesis of both CRC and HCC.

If alcohol have already caused significant genetic and epigenetic alterations in the liver of ALD patients with CRC, then the diagnosis of CRC may be regarded as a surrogate marker for the risk of developing subsequent HCC in ALD. It can be argued that ALD patients who developed CRC might receive more frequent imaging studies, resulting in an increased chance of HCC detection (lead-time bias). If this is the case, then HCC stages may be less advanced in ALD patients with CRC compared to patients who developed HCC without preceding CRC, which is not the case in our data: the size of HCC was not significantly different between patients with and without CRC. Further studies will be needed, however, to elucidate the mechanisms underlying the association between CRC and HCC in ALD.

Liver cirrhosis^38^, elevated baseline AFP level^39^ and low platelet counts^25,40^ have been reported as significant predictors of HCC in ALD, and our data are line with previous reports. Alcohol intake amount was associated with the risk of HCC in phase I case-control analysis and univariate cohort study, but it was not an independent predictor of HCC in multivariate cohort analysis, probability due to multicollinearity with CRC (Table 3).

HCC surveillance has not been routinely recommended for patients with ALD, due to the widely variable range of its risk and relatively lower contribution of HCC to the mortality of alcoholic hepatitis and/or cirrhosis^13,41^. Our cohorts also showed relatively low incidence of HCC (33/100,000 person-years), which is consistent with the previous reports^38^. In contrast, diagnosis of CRC increased the risk of developing HCC by more than 12 folds in univariate analysis. Although CRC develops infrequently in patients with ALD, we suppose that HCC surveillance should be considered when CRC, even with early-stage, is detected in those with ALD. Since patients with no risk factor may have very low risk of developing HCC (Fig. 3), regular HCC surveillance may not be warranted^6^, whereas those with CRC, especially with additional risk factor(s), may have significantly higher risk of HCC and regular HCC surveillance may be justified. However, further external validation needs to be conducted to evaluate the clinical utility of our risk stratification and the pragmatic strategy of individualized HCC surveillance in ALD.

We excluded patients with Child-Pugh class C (n = 6; 1 with HCC and 5 without HCC) in Phase I analysis because the history of CRC might be less accurate in this group (limited colonoscopic examinations due to grave hepatic prognosis and/or presence of ascites). However, inclusion of these 6 patients for logistic regression analysis has not changed the results of Table 2 (shown in Supplementary Table 1 and Table 2).

Smoking history had the greatest odds ratio among the predictors in Phase I analysis, but showed insignificant hazard ratio in Phase II Cox analysis. We believe that the number of HCC was larger in Phase I analysis (24 vs. 445), so that type 2 error (beta error) occurred for smoking in Phase II analysis, considering the recent reports supporting the carcinogenic potential of smoking in HCC^42,43^. Since most CRC patients were smokers (23/24, Table 3), further data would be needed to determine whether non-smokers with CRC pose higher risk for HCC.

Our study has several limitations. First, due to the case-control and retrospective cohort design, biases cannot be completely avoided. We do not suppose that the probability of recall bias may be high, because CRC is not a casual history. We also employed an electronic health record system in order to include every eligible patient without omission^21,22,44^. Despite these efforts, our findings still need to be externally validated by prospective cohort studies. Second, relatively small number of patients had CRC compared to CRA in our cohort. However, since the CRC history is a significant predictor of HCC regardless of cirrhosis, these patients may need to receive enhanced surveillance for HCC. Again, validation in larger-scale studies is still needed with more CRC cases. Third, our multivariate analyses did not include the stage of fibrosis, which may be a major determinant of HCC risk. Considering the invasiveness of biopsy, however, we believe that our non-invasive risk factors can be applied to ALD patients without histologic data in the real-world practice settings. Liver stiffness measurement (LSM) is useful in estimating the risk of HCC development in patients with chronic viral hepatitis^45^, and the predictive role of LSM may warrant further validation in patients with ALD^45–47^.

In conclusion, the results of this cross-sectional and cohort study indicate that ALD patients with CRC are more likely to develop HCC than patients without history of CRC. CRC, liver cirrhosis, elevated AFP level, and thrombocytopenia independently predict the risk for HCC in patients with ALD.

## Supplementary information

Supplementary Tables

## Data Availability

Data may be available on request after review of the request by our institutional review boards.
