# Supplementary material for: Development of colorectal cancer predicts increased risk of subsequent hepatocellular carcinoma in patients with alcoholic liver disease: case-control and cohort study
Source: Sci Rep. 2019 Mar 1;9:3236. doi: 10.1038/s41598-019-39573-9 (PMC6397178; doi:10.1038/s41598-019-39573-9)
Supplement: Supplementary file 1 — Supplementary Tables [file 41598_2019_39573_MOESM1_ESM.docx]

**Supplementary information**

**Development of colorectal cancer predicts increased risk of subsequent hepatocellular carcinoma in patients with alcoholic liver disease: case-control and cohort study**

Won Kim*, MD, PhD^1,3^, Dongjae Jeong*, BS^1^, Jungwha Chung, MD^2^, Donghyeon Lee, MD^1,3^**,** Saekyoung Joo, MD^1,3^, Eun Sun Jang, MD, PhD^2^, Yoon Jin Choi, MD, PhD^2^, Hyuk Yoon, MD, PhD^2^, Cheol Min Shin, MD, PhD^2^, Young Soo Park, MD, PhD^2^, Sook-Hyang Jeong, MD, PhD^1, 2^, Nayoung Kim, MD, PhD^1, 2^, Dong Ho Lee, MD, PhD^1, 2^ and Jin-Wook Kim, MD, PhD^1, 2^

^1^Department of Medicine, Seoul National University College of Medicine, Seoul, Republic of Korea

^2^Department of Medicine, Seoul National University Bundang Hospital, Seongnam, Republic of Korea

^3^Department of Internal Medicine, Seoul Metropolitan Government Seoul National University Boramae Medical Center, Seoul, Republic of Korea

*Both authors contributed equally to this work.

**2 supplementary tables.**

**Supplementary Table 1. Case-control analysis: characteristics of alcoholic liver disease patients with and without hepatocellular carcinoma**, **including Child-Pugh class C patients**

|  | ALD patients with HCC cases  (n = 446) | Control ALD patients without HCC  (n = 1,219) | *P* value |
| --- | --- | --- | --- |
| Age, years | 64 (15) | 49 (14) | **<.001** |
| Male | 411 (92) | 1,163 (95) | **.01** |
| Drinking amount (> 80g/day of ethanol) | 315 (71) | 714 (59) | **<.001** |
| Current or ex-smoker | 444 (99.8) | 1,171 (96.1) | **<.001** |
| Prior history of CRC | 24 (5.4) | 33 (2.7) | **.008** |
| AFP (ng/mL) | 5.4 (23.3) | 3.0 (1.9) | **< .001** |
| Albumin (g/dL) | 3.8 (1.0) | 4.5 (0.5) | **<.001** |
| Bilirubin (mg/dL) | 1.1 (1.0) | 1.1 (0.6) | .260 |
| AST (U/L) | 47 (45) | 35 (27) | **<.001** |
| ALT (U/L) | 32 (31) | 41(34) | **<.001** |
| GGT (U/L) | 121 (205) | 77 (102) | **<.001** |
| Platelet count, ×10^9^/L | 162 (121) | 224 (106) | **<.001** |
| Prothrombin time (INR) | 1.10 (0.25) | 1.01 (0.09) | **<.001** |

Continuous variables are expressed as the median (interquartile range) and categorical variables are presented as numbers (%).

Laboratory data were values at the index date (confirmation of presence/absence of HCC).

*P* values were calculated by Wilcoxon rank sum test or χ^2^ test for continuous and categorical variables, respectively.

CRC, colorectal cancer.

**Supplementary Table 2. Odds ratio for HCC in the phase I case-control analysis (n = 1,665 including Child-Pugh class C patients)**

|  | Unadjusted OR (95% CI) | Adjusted OR (95% CI) |
| --- | --- | --- |
| Age (> 35 years) | **3.44 (1.65 – 7.21)**** | **3.46 (1.64 – 7.30)**** |
| Male | **0.57 (0.37 – 0.88)**** | **0.54 (0.35 – 0.85)**** |
| Drinking amount (> 80 g ethanol/day) | **1.70 (1.35 – 2.15)*** | **1.73 (1.36 – 2.19)*** |
| Current or ex-smoker | **4.17 (1.71 – 10.09)*** | **18.5 (2.53 - 135.1)**** |
| Prior history of CRC | **2.04 (1.19 – 3.50)*** | **1.83 (1.06 – 3.15)**** |

**P* <.001, ** *P* <.05

CRC, colorectal cancer; OR, odds ratio
